# Metabolic syndrome and Visceral Adiposity Index in non-functional adrenal adenomas

**DOI:** 10.20945/2359-3997000000547

**Published:** 2022-12-01

**Authors:** Savas Karatas, Yalcin Hacioglu, Selvihan Beysel

**Affiliations:** 1 Istanbul Research and Education Hospital Endocrinology and Metabolism Department Istanbul Turkey Istanbul Research and Education Hospital, Endocrinology and Metabolism Department, Fatih, Istanbul, Turkey; 2 Istanbul Research and Education Hospital Family Medicine Department Istanbul Turkey Istanbul Research and Education Hospital, Family Medicine Department, Fatih, Istanbul, Turkey; 3 Afyon Kocatepe University Endocrinology and Metabolism Department Afyon Turkey Afyon Kocatepe University Endocrinology and Metabolism Department, Afyon, Turkey

**Keywords:** Visceral Adiposity Index, adrenal adenoma, metabolic syndrome

## Abstract

**Objective:**

We aimed to identify metabolic dysfunction in non-functioning adrenal adenomas (NFAAs) and Visceral Adiposity Index (VAI) predictability in the practical estimation of metabolic syndrome (MetS) in NFAAs.

**Subjects and methods:**

134 NFAA patients and 68 control subjects matched for age, sex, and body mass index (BMI) were included in the study. After physical, biochemical, and endocrine evaluation, IDF and NCEP ATP III criteria were used to determine MetS. HOMA-IR and VAI were calculated for both study group subjects.

**Results:**

MetS was significantly higher in the NFAA patients. The incidence of MetS by IDF and NCEP criteria was 52.9%,48.5% in the NFAI and 32.3%,30.8% in the control group (p < 0.01, p = 0.02). The risk of MetS was increased in NFAA (75.6 *vs.* 24.4%, p = 0.017, OR = 1.34, 95% CI = 1.06-1.68). Glucose, HOMA IR, hypertension, and VAI were significantly increased in NFAA patients. The risk of MetS was independently associated with high VAI (79.2 *vs.* 20.8%, p = 0.001, OR = 2.22; 95% CI = 1.70-2.91).

**Conclusion:**

MetS, insulin resistance, and VAI are more prevalant in NFAA patients than in healthy individuals. VAI can be used with high specificity to estimate MetS in NFAA patients.

## INTRODUCTION

An adrenal incidentaloma (AI) has been defined as a randomly found out mass greater than 1 cm in size during abdominal imaging usually performed for some other reason (1). Increased opportunity to reach imaging procedures has made AI diagnosis more common than in the past. The prevalence of AI has increased to 4% in middle age and 10% in the older people (2). In a recent analysis of 603 patients, the prevalence was 7.3% (3). Following an endocrine exclusion procedure, AIs lacking secretory capacity are defined as non-functioning adrenal adenomas (NFAAs), which account for 71%-84% of AIs (1,4,5). Until now, NFAAs have generally been considered clinically insignificant. European Society of Endocrinology/European Network for the Study of Adrenal Tumors guidelines recommended clinical, radiological, and hormonal follow-up (1), however, recent studies have shown that NFAAs have metabolic properties. These were a few studies that have examined the metabolic features of NFAAs and found increased insulin resistance, altered glucose tolerance, increased blood pressure, waist circumference, and increased risk of diabetes (3,6,7).

A new mathematical model, the Visceral Adiposity Index (VAI), was developed by Amato and shows a strong correlation with visceral adipose tissue measured directly by magnetic resonance imaging (8). VAI is a widespread area of importance and research interest, and the results of related studies showed that high levels of VAI are associated with increased cardiovascular events, glycaemic disorders and metabolic syndrome (MetS) (9,10,11).

There are few studies on the relationship between metabolic dysfunction and NFAAs. Our aim was to investigate the physical metabolic characteristics of NFAAs and to explore the predictability of VAI of NFAAs with metabolic risk. This study will provide new data on the metabolic risk and characteristics of NFAA patients and contribute to the literature with its patient population.

## SUBJECTS AND METHODS

### Participants

One hundred and seventy-five consecutive NFAA patients examined between October 2018 and October 2020 in Istanbul Research and Educational Hospital Endocrinology Outpatient Clinic were included in this retrospective and cross-sectional study. Sixty-eight healthy control subjects who had normal adrenal glands on computerized tomography (CT) scan were selected from the hospital database.

The inclusion criteria for NFAA were: 1. For subjects with unilateral or bilateral adrenal adenoma larger than 1 cm and smaller than 4 cm, apparent features of benign adrenal adenoma confirmed by a radiologist (homogeneous, well bordered, regular shape) detected on CT-scan or abdominal MRG. 2. Subjects who have no typical signs of hypercortisolism (abdominal stria, moon face, buffola hump, etc.), whose serum cortisol level is suppressed; <50 nmol/L (<1.8 mcg/dL), after 1 mg of dexamethasone or when the dexamethasone test is higher than 50 nmol/L, (>1.8 mcg/dL), whose serum cortisol level is suppressed after 2 mg of dexamethasone test and whose free cortisol level is normal over 24 hours (1). 3. Normal upright plasma aldosterone/renin ratio in concomitant hypertension and/or hypokalemia. 4. Normal plasma levels of free metanephrine, normetanephrine, and/or urinary fractionated free metanephrine, normetanephrine in urine. 5. euthyroidism, no chronic diseases affecting hormone tests. 6. No medication use that may affect dexamethasone clearance.

The exclusion criteria for NFAA patients and the control group were: 1. pregnancy, estrogen use, breastfeeding, <18 age; 2. presence of chronic diseases that may affect physical, metabolic values and hormonal tests (hyperthyroidism, hypothyroidism, renal failure, liver failure, cancer, chronic infections, critically ill patients); 3. medication or drug use that may affect physical values, metabolic values, and hormone tests.

After exclusion criteria, one hundred and thirty-four NFAA patients were eligible for the study. Sixty-eight subjects matched for age, sex, and body mass index (BMI) were included in the study as controls.

### Anthropometric measurements

Height and weight were measured by standard methods, and body mass index (BMI) was calculated. BMI was calculated by dividing weight (in kg) by the square of height (in m). Waist circumference was measured using a measuring tape, with measurements taken midway between the ribs’ bottom and the iliac crest on a horizontal plane.

Systolic and diastolic blood pressure were measured by sphingomanometry, and hypertension was defined according to the Joint National Committee Eight (JNC VIII). Patients with a systolic blood pressure of ≥140 mmHg and diastolic blood pressure of ≥90 mmHg were defined as hypertensive. When defining metabolic syndrome criteria, the related criterion was used. The presence of hypertension and the use of antihypertensive medication were also recorded.

### Biochemical analysis

All biochemical tests were performed after 12 hours of fasting in the morning. Fasting blood glucose, HDL cholesterol, triglycerides, LDL cholesterol, and insulin levels were measured in the same hospital laboratory.

Diabetes diagnosis was made according to the criteria of American Diabetes Association (ADA) (12); 75 g OGTT was performed at fasting glucose levels between 100-126 mg/dL. Previous diabetes history was also recorded.

### Hormonal analysis

Endocrine examination was performed in all NFAA patients. Basal cortisol levels were measured at 08:00-09:00 am. Overnight 1 mg dexamethasone was administered at 23:00 hrs, and serum cortisol level was measured at 08:00-09:00 hrs the next morning. The cut-off value for serum cortisol, which was less than 1.8 mcg/dL, was used to exclude hypercortisolism (1). Plasma aldosterone and renin activity were measured to exclude hyperaldosteronism. Plasma renin aldosterone cut-off value was taken as 1.6 pmol/L/min according to European Endocrine Society Guideline (13). Urinary metanephrine and normetanephrine levels were measured to exclude pheochromocytoma. The glucose oxidase/peroxidase method was used to determine fasting glucose levels. High-density lipoprotein cholesterol (HDL-C) and triglycerides (TG) were measured by spectrophotometry using enzymatic colorimetric assays. The Friedewald formula was used to determine low-density lipoprotein cholesterol (LDL-C). Cortisol levels were measured by the chemiluminescence method (Unicel Dxl 800 Immunoassay System, Beckman-Coulter Inc., USA). Aldosterone and renin levels were determined by the chemiluminescence enzyme immunoassay method. The 24-h urinary catecholamines were determined by liquid chromatography/tandem mass spectrometry. The homeostasis model assessment of insulin resistance (HOMA-IR) was measured by the formula [fasting glucose (mg/dL) × fasting insulin (μU/mL)/405] (14).

### Definitions and other parameters

MetS frequency was calculated using the National Cholesterol Education Program Adult Treatment Panel III (NCEP ATPIII, American Heart Association Reviewed), and International Diabetes Federation criteria. NCEP ATP III criteria included; 1) waist circumference >88 for women, >102 for men; 2) fasting blood glucose ≥100 mg/dL or antihyperglycemic medication; 3) triglycerides ≥1.7 mmol/L (150 mg/dL) and/or any anti hypertriglyceridemia medication; 4) decreased HDL cholesterol (<40 mg/dL for men, <50 mg/dL for women); 5) ≥130 mmHg systolic or ≥85 mmHg diastolic blood pressure or antihypertensive treatment. Three of these criteria were defined as metabolic syndrome (15). IDF criteria included; 1) central obesity men ≥94 cm, women ≥80 cm (absolutely required, and two of the following items); 2) fasting blood glucose ≥100 mg/dL or antihyperglycemic medication; 3) triglycerides ≥1.7 mmol/L (150 mg/dL) and/or any anti hypertriglyceridemia medication; 4) decreased HDL cholesterol (<40 mg/dL for men, <50 mg/dL for women); 5) ≥130 mmHg systolic or ≥85 mmHg diastolic blood pressure or antihypertensive treatment (16). VAI was calculated according to the following formula: Men: VAI = (WC /(39.68 + (1.88 × BMI))) × (TG /1.03) × (1.31/HDL-C) Women: VAI = (WC /(36.58 + (1.89 × BMI))) × (TG /0.81) × (1.52/HDL-C) (TG and HDL-C were expressed in mmol/L) (8).

The protocol was approved on November.11.2019 by the Istanbul Research and Educational Hospital (Istanbul, Turkey) ethics committee under Protocol n° 2026.

### Statistical analysis

Statistical analysis was performed using SPSS 22.0 (SPSS Inc., Chicago, IL, USA). Chi-square test or Fischer's test were used to measure the categorical parameters. Student's t-test was used for two-group comparisons of variables with normal distribution and comparison of descriptive statistical methods (mean, standard deviation, median, frequency, ratio, minimum, and maximum) and quantitative data. Mann-Whitney U test was used accordingly for two-group comparisons. Pearson's correlation test or Spearman test was used for calculating correlations. Logistic regression analysis was performed to analyze factors affecting the frequency of metabolic syndrome. A receiver operator curve analysis (ROC) was performed to evaluate VAI scores according to metabolic syndrome status. Logistic regression analysis was used to determine the predictability of VAI for Mets. Significance was assessed at p 0.05.

## RESULTS

One hundred and seventy five consecutive NFAA patients examined at the Endocrinology Outpatient Department of Istanbul Research and Educational Hospital between October 2018 and October 2020 were included in this retrospective and cross-sectional study. Sixty-eight healthy control subjects whose adrenal glands were normal on computed tomography (CT) were selected from the hospital database.

Fifty-three and 5% of the adenomas were left-sided, 31,3% were right-sided, 12,7% were bilateral. The mean adenoma size was 21.58 ± 7.5 mm. NFAA patients and control patients were age, gender, and BMI matched (p > 0,05) (Table 1)

**Table 1:** General characteristics of study groups

	NFAD (n = 134)	Control (n = 68)	p
Gender (female)	67.9% (n = 91)	64.7% (n = 44)	0.65
Age (years)	54.3 ± 8.9 (mean)	52.2 ± 10.8 (mean)	0.15
Body weight (kg)	85.0 ± 16.4 (mean)	84.5 ± 14.4 (mean)	0.81
BMI (kg/m^2^)	32.4 ± 5.9 (mean)	31.7 ± 5.5 (mean)	0.39
WC (cm)	103.0 ± 12.9 (mean)	102.9 ± 11.2 (mean)	0.95

NFAA: non-functioning adrenal adenoma; M: male; F: female; BMI: body mass ındex; WC: waist circumference

NFAA patients had higher mean fasting glucose values (105.9 ± 18.8 *vs.* 97.8 ± 12.3 mg/dL, p < 0.01). Postprandial glucose, LDL cholesterol, TG and HDL-C levels were similar between groups (p > 0.05, Table 2). NFAA patients had a higher incidence of hypertension (TA > 130/85 or history of hypertension) (Table 3, p < 0.05). Insulin levels were not significant and mean HOMA IR was significantly higher in the NFAA group (Table 2, p = 0.06 and 0.02, respectively). Prevalance of MetS according to IDF criteria (52.9% *vs.* 32.2%) and NCEP ATPIII criteria (48.5% *vs.* 30.8%) was higher in NFAA group (Table 4, p < 0.01 and p = 0.02 respectively). According to IDF (52.9% vs 32.3%, p<0.01) and NCEP ATP III (48.5 % vs 30.2 %, p=0.02) criteria MetS frequency was increased in NFAA group (Table 5). MetS risk was increased with NFAA (75.6 *vs.* 24.4%, p = 0.017, OR = 1.34, 95% CI 1.06-1.68).

**Table 2:** Laboratory evaluation of the NFAA patients and healthy subjects

	NFAA (n = 134)	Control (n = 68)	p
Glucose (mg/dL)	105.9 ± 18.8	97.8 ± 12.3	**<0.01**
PPG (mg/dL)	123.4 ± 41.7	113.0 ± 27.5	0.11
LDL (mg/dL)	135.9 ± 35.9	134.3 ± 36.8	0.78
Triglyceride (mg/dL)	131.8 ± 61.0	130.2 ± 49.1	0.85
HDL-C (mg/dL)	52.6 ± 11.8	54.6 ± 13.0	0.27
Insulin (μIU/dL)	12,5 ± 10,8	10.1 ± 5.4	0.06
HOMA-IR	3.44 ± 3.2	2.39 ± 1.3	**0.02**

NFAA: non-functioning adrenal adenoma; PPG: post-prandial Glucose; HOMA IR homeostasis model assessment-insulin resistance; LDL-C: LDL cholesterol; HDL-C: HDL cholesterol. Significant p values were shown in bold.

**Table 3:** Prevalence of metabolic syndrome criteria according to IDF

Criteria	NFAA (n: 134)	Control (n: 68)	p
FBG ≥ 100 (mg/dL) or type 2 diabetes story	57.4% (n = 77)	42.6% (n = 29)	**0.04**
WC (≥80 for female, ≥94 for male (mg/dL)	93.2% (n = 125)	92.6% (n = 63)	0.82
BP ≥ 130/85 or HT story	44.0% (n = 59)	22.1% (n = 15)	**0.02**
TG ≥ 150 (mg/dL) or treatment story	36.5% (n = 49)	30.7% (n = 16)	0.06
HDL-C < 40 for male, < 50 for female (mg/dL) or treatment story	34.3% (n = 46)	26.9% (n = 17)	0.18

FBG: fasting blood glucose; WC: waist circumference; BP: arterial blood pressure; HT: hypertension; TG: trlyglyceride; HDL-C: HDL cholesterol. Significant p values were shown in bold.

**Table 4:** Prevalence of metabolic syndrome criteria according to NCEP-ATP III

Criteria	NFAA (n: 134)	Control (n: 68)	p
FBG ≥ 100 mg/dL or antihyperglycemic medication	56.7% (n = 76)	42.6% (n = 29)	**0.04**
WC ≥ 88 for women, ≥ 102 for men	82.0 (n = 110)	80.8 (n = 55)	0.76
TG ≥ 1.7 mmol/L (150 mg/dL) and/or any anti hypertriglyceridemia medication	36.5% (n = 49)	30.7% (n = 16)	0.06
Decreased HDL-C (<40 mg/dL for men, <50 mg/dL for women)	34.3% (n = 46)	26.9% (n = 17)	0.18
BP ≥ 130/85 or antihypertensive treatment	42.5% (n = 57)	20.5% (n = 14)	**0.02**

FBG: fasting blood glucose; WC: waist circumference; TA: arterial blood pressure; BP: blood pressure; TG: trlyglyceride; HDL-C: HDL cholesterol. Significant p values were shown in bold.

**Table 5:** Comparison of groups according to metabolic syndrome frequency and VAI

	NFAI (n = 134)	Control (n = 68)	p
MetS IDF	52.9% (n = 71)	32.3% (n = 22)	**<0.01**
MetS NCEP	48.5% (n = 65)	30.8% (n = 21)	**0.02**
VAI (mean)	4.82 ± 2.45	2.82 ± 1.45	**0.01**

MetS IDF: metabolic syndrome according to Internationa Diabetes Syndrome Criteria; MetS NCEP: Metabolic syndrome according to National Cholesterol Education Programme ATP III criteria; VAI: Visceral Adiposity Index. Significant p values were shown in bold.

**Figure 1:**
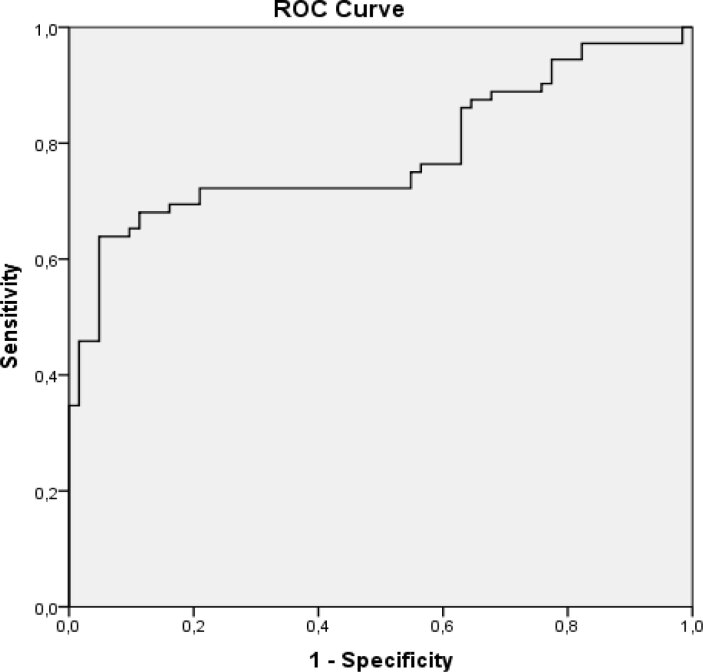
ROC Curve for VAI and MetS.

VAI was increased in NFAA with MetS compared with NFAA without MetS (4.86 ± 2.61 versus 2.55 ± 1.15, p < 0.01). The area under the curve (AUC) for VAI to detect MetS (according to IDF) was 0.781. We calculated a cut-off value of 4.16 for VAI (sensitivity: 63.4%, specificity: 93.7%) (Figure 1).

AUC: 0.781; VAI: Visceral Adiposity Index; Mets: metabolic syndrome.

After using a model of age, sex, 1 mg DST, VAI and HOMA-IR; VAI was a high predictor of MetS in NFAAs (β = 3.54, p < 0.001, 95% CI = 2.52-8.59). Cox&Snell R2 = 0.472. The risk of MetS was independently associated with high VAI (79.2 *vs.* 20.8%, p < 0.001, OR = 2.22; 95% CI 1.70-2.91). There was no significant correlation between VAI and cortisol after 1 mg DST (r = 0.118, p = 0.20) HOMA-IR and VAI had shown a higher correlation (r = 0.173, p = 0.02).

## DISCUSSION

In this study, we have found Mets higher in NFAAs according to IDF; compared to age, gender and BMI adjusted healthy subjects. (52.9% *vs.* 32.3%). All metabolic syndrome criteria were higher in the NFAA group and increased glucose and blood pressure were significantly more common in NFAA patients. We found significantly increased visceral adiposity in NFAA patients.

According to several studies, MetS similar to our study was found more frequently in NFAA, but these studies were conducted with smaller groups of patients, some of them investigated only one or more components of metabolic syndrome. In a study with 74 NFAA patients, an increased MetS ratio was also found.^7^ The ratio of MetS, diabetes and HT was higher in this study, however, the average age was also higher than in our groups.

The most common criterion for metabolic syndrome after waist circumference was hyperglycaemia and history of diabetes in the NFAA group. Similar to our study, Krzyżewska and cols. found that hyperglycaemia and diabetes were more common in NFAA patients (17). In another study from Japan, glucose intolerance in NFAA was found to be equivalent to a functioning adrenal adenoma (18). In a longitudinal study, NFAA patients were found to have a higher risk of diabetes. (19).

Hypertension and HOMA-IR levels were significantly higher in the NFAA group, but TG and HDL levels did not differ significantly. In another study by Dalmazi, the incidence of hypertension was also higher in NFAA compared to healthy subjects (20). Peppa and cols. demonstrated higher cholesterol, HOMA IR and hypertension levels in 29 patients (21). However, the mechanisms underlying these increased cardiovascular risks are not clear.

Initially, it was postulated that adrenal incidentaloma was a new manifestation of the metabolic syndrome that might result from insulin-mediated stimulation (22). One possible mechanism of these increased metabolic abnormalities in NFAA was increased cortisol secretion, which could not be measured by routine laboratory tests. Increased urinary cortisol metabolites in NFAA have been suggested as possible metabolic effects (23,24). In contrast, a recent study did not find increased cortisol metabolites in NFAA (25). Cortisol may act as an agonist at glucocorticoid and mineralocorticoid receptors. Tzanela and cols. found that the BCL1 GR polymorphism did not affect the frequency of cortisol-associated comorbidities in dexamethasone suppression test-negative NFAAs (26).

VAI, a mathematical model developed by Amato and cols. that shows a significant correlation between superficial area and visceral adipose tissue volume (8). The formula VAI includes three of the MetS criteria (TG, HDL and WC). It has been shown to be the most excellent discriminating factor for metabolic syndrome in men and women in a large cohort study (27). Similarly, a Chinese prospective study of 3461 subjects and a Qatari prospective study of 1,103 subjects found that VAI is an independent risk factor for type 2 diabetes (28,29). Also Bagyura and cols. found that higher VAI scores were associated with increased coronary calcium scores in male patients (30). Although several studies have been conducted showing increased cardiovascular risk in NFAA, there is only one study that measured visceral adipose tissue in NFAA and found equivalent VAI between NFAAs and a BMI-matched control group (31). Another study that found increased epicardial fat thickness in NFAAs (32).

In conclusion, this study was consistent with several previous studies indicating increased cardiovascular risk in NFAAs. Increased MetS, increased VAI, VAI's predictability of MetS and its relationship with insulin resistance were the cardinal findings of the current study.

Limitations of our study were; its cross-sectional design, the fact that we were not able to assess nutritional and exercise habits and couldn't directly measure visceral adipose tissue in the study groups, and the fact that we were not able to use the gold standard technique for measuring insulin resistance. However, we have investigated all the metabolic syndrome criteria in the study. A key strength of the research lies within the fact that group size was not relatively small.

In conclusion, MetS, insulin resistance, diabetes, hypertension and VAI are increased in NFAA patients compared to healthy individuals. VAI can be used with high specificity to assess metabolic syndrome in NFAA patients.
